# Development and Remodeling of the Vertebrate Blood-Gas Barrier

**DOI:** 10.1155/2013/101597

**Published:** 2012-12-27

**Authors:** Andrew Makanya, Aikaterini Anagnostopoulou, Valentin Djonov

**Affiliations:** ^1^Institute of Anatomy, University of Bern, Baltzerstrasse 2, 3000 Berne, Switzerland; ^2^Department of Veterinary Anatomy & Physiology, University of Nairobi, Riverside Drive, P.O. Box 30197, Nairobi 00100, Kenya

## Abstract

During vertebrate development, the lung inaugurates as an endodermal bud from the primitive foregut. Dichotomous subdivision of the bud results in arborizing airways that form the prospective gas exchanging chambers, where a thin blood-gas barrier (BGB) is established. In the mammalian lung, this proceeds through conversion of type II cells to type I cells, thinning, and elongation of the cells as well as extrusion of the lamellar bodies. Subsequent diminution of interstitial tissue and apposition of capillaries to the alveolar epithelium establish a thin BGB. In the noncompliant avian lung, attenuation proceeds through cell-cutting processes that result in remarkable thinning of the epithelial layer. A host of morphoregulatory molecules, including transcription factors such as Nkx2.1, GATA, HNF-3, and WNT5a; signaling molecules including FGF, BMP-4, Shh, and TFG-**β** and extracellular proteins and their receptors have been implicated. During normal physiological function, the BGB may be remodeled in response to alterations in transmural pressures in both blood capillaries and airspaces. Such changes are mitigated through rapid expression of the relevant genes for extracellular matrix proteins and growth factors. While an appreciable amount of information regarding molecular control has been documented in the mammalian lung, very little is available on the avian lung.

## 1. Introduction

The pulmonary blood-gas barrier (BGB) performs the noble role of passive diffusion of gases between blood and a common pool that delivers the air to the exchanging structures. The BGB is a paradoxical bioengineering structure in that it attains remarkable strength while at the same time remaining thin enough to allow gas exchange. The two aspects of the BGB important for efficient exchange are thinness and an extensive surface area. Additionally, the barrier needs to be strong to withstand stress failure, as may occur due to increased blood capillary pressure during exercise [1]. The presence of collagen IV within the basement membranes is associated with the remarkable strength characteristic of the BGB [2].

In vertebrates, the design of the BGB is governed by many factors, including evolutionary status, gas exchange medium, and level of physiological activity.

**Figure 1:**
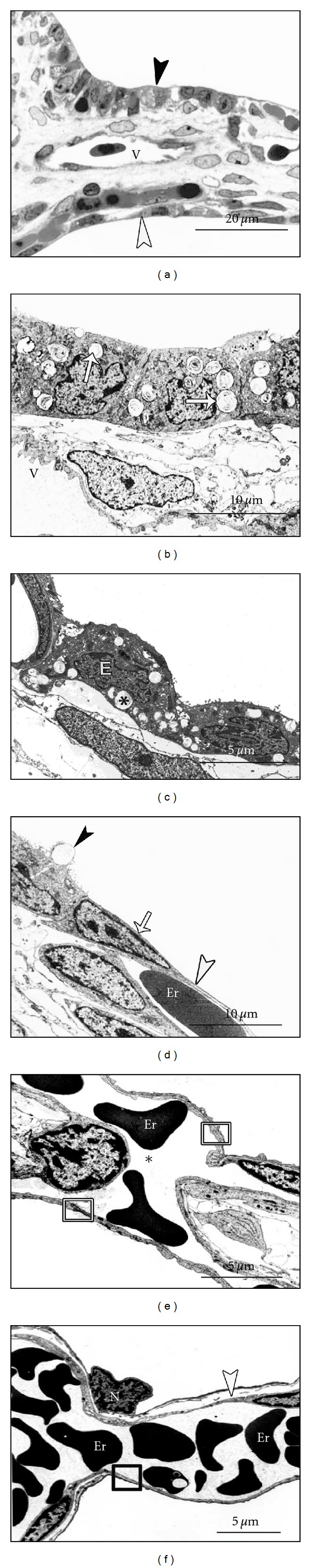
Micrographs showing the changing pulmonary epithelium in the developing quokka lung. (a) At the canalicular stage, both cuboidal (closed arrowhead) and squamous epithelium (open arrowhead) are present. At the centre of the thick interstitium is a large blood vessel (V). (b) The cuboidal epithelium comprises of cells well-endowed with lamellar bodies (white arrows). These cells notably lack microvilli and may be described as pneumoblasts with a potential to form either of the two definitive alveolar pneumocytes (AT-I and AT-II). Note the large blood vessel (V) below the epithelium. ((c) and (d)) During the saccular stage the epithelial cells (E) possess numerous lamellar bodies (asterisk) and have become low cuboidal in the process of conversion to AT-I cells. AT-II cells converting to AT-I pneumocytes appear to do so by extruding entire lamellar bodies (closed arrowhead in (d)) and flattening out (arrow). Notice the already formed thin BGB (open arrowhead) and an erythrocyte (Er) in the conterminous capillary. ((e) and (f)) Immature interalveolar septa (E) are converted to mature ones through fusion of capillary layers (asterisk in (e)) and reduction in interstitial tissue. The process starts during the alveolar stage and continues during the microvascular maturation stage. Notice the thin BGB (square frames) and the thick side of the BGB in adults (open arrowhead in (f)). The Erythrocytes (Er) and a nucleus (N) belonging to a AT-I cell are also shown. (a)–(c) are from [11], (d) is from [12] while (e) and (f) were obtained from [13], all with permission from the publishers.

The BGB has been most refined in avians whereby it is reputed to be largely uniform on both sides of the capillary and is generally 2.5 times thinner than that in mammals [2]. In the developing mammalian lung at saccular stage, interairspace septa have a double capillary system [3], and hence only one side of the capillary is exposed to air. Such are generally referred to as immature septa. In mammals, this double capillary system is converted to a single one [3] except in some primitive ones such as the naked mole rats (*Heterocephaus glaber*), where it persists in adults [4]. In adult mammals, the BGB occurs in two types, a tripartite thinner one that comprises the alveolar epithelium, which is separated from the capillary endothelium by a basal lamina, (Figure 1) and a thicker one where an interstitium intervenes between the epithelial basal lamina and the endothelial basal lamina [19]. In ectotherms generally the immature septa with a double capillary system preponderate [5].

Generally, the vertebrate lung develops from the ventral aspect of the primitive gut, where the endodermal layer forms a laryngotracheal groove, which later forms the lung bud [6]. This occurs at about embryonic day 9 (E9) in mice, E26 in humans [7], and E3-E4 in the chick [8]. In mammals, there is dichotomous branching of the primitive tubes of the early lung leading to formation of the gas exchange units. In birds, the initial step in dichotomous branching gives rise to the primary bronchi, which proceed to form the mesobronchi. Development of the secondary bronchi, however, does not appear to follow the dichotomous pattern since certain groups of the secondary bronchi arise from prescribed areas and have a specific 3D orientation [9, 10]. While a wealth of the literature exists on the mammalian lung development, the picture on the avian is just beginning to emerge. In contrast, development in ectotherms appears to have been ignored by contemporary investigators.

## 2. Structure of the Blood-Gas Barrier in Vertebrates

The basic components of the blood-gas barrier are the epithelium on the aerated side, the intermediate extracellular matrix (ECM), and the capillary endothelium on the perfused side. In mammals, the thickest component of the BGB is the ECM. Calculations from data provided by Watson et al. [14] indicate that the ECM takes 42% and 40% of the entire thickness in the horse and dog, respectively, whereas the epithelium and the endothelium take almost equal proportions at about 28–30%. Unlike in mammals, the interstitium in the avian lung is the thinnest component of the BGB at 17%, while the endothelium is the thickest at 51% [15]. Additionally, the layers of the BGB in the chicken lung are remarkably uniform in thickness over wide regions. The chicken ECM measures about 0.135 *μ*m (arithmetic mean thickness) and mainly comprises of fused basement membranes of the epithelium and endothelium. In ectotherms, the ECM is abundant and lies between the two capillary layers, as well, as within the BGB, and hence they have a thicker BGB than either mammals or birds.

The thickness of the blood-water/air (tissue) barrier increases from fish, amphibians, reptiles, and mammals to birds [2, 5]. In humans, the thin side has a thickness of 0.2-0.3 *μ*m and covers approximately half of the alveolar wall [16]. It is made up of the fused basement membranes of the epithelial and endothelial layers and is the critical structure for pulmonary gas exchange and stress failure. In contrast, the thick side also contains interstitial cells, such as fibroblasts and pericytes, as well as type I collagen fibers that are important in the scaffold support of the lung. This thick side measures up to 1 *μ*m or more in humans [17] and may be as little as 0.1 *μ*m or less in some domestic mammals [18]. The tensile strength of the basement membrane comes from type IV collagen, which is synthesized by both epithelial and endothelial cells, and in smaller amounts by other mesenchymal cells. A detailed review of the structure and remodeling of the BGB was provided by West and Mathieou-Costello [19].

Amongst vertebrates, the lung is more specialized in endotherms (mammals and birds) compared to ectotherms (fish, amphibians, and reptiles). The barrier is thicker in fish gills but relatively thin in the lung of air-breathing fishes. In the gills of the air-breathing Amozonian fish (*Arapaima gigas*), BGB is 9.6 *μ*m, while at the swim bladder the harmonic mean thickness of the BGB is 0.22 *μ*m [20]. In lungs of amphibians and reptiles, it is thinner than in fish gills. In amphibians, it ranges from 1.21 *μ*m in the South African clawed toad (*Xenopus laevis*) [21] to 2.34 *μ*m in the common newt (*Triturus vulgaris*) [21]. In reptiles, the BGB is generally much smaller than in amphibians, and the range is also narrower. The smallest recorded maximal harmonical mean thickness was in the red-eared turtle (*Pseudemys scripta*) at 0.46 *μ*m [21], while the highest was in the Nile crocodile (*Crocodylus niloticus*) at 1.4 *μ*m [22]. Among vertebrates, the thinnest BGB has been encountered in birds and highly active mammals. In the African rock martin (*Ptyonoprogne filigula),* it measures 0.09 *μ*m, while in the violet-eared hummingbird (*Colibri coruscans),* it is 0.099 *μ*m [5]. Specialization of the lung amongst mammals appears to be most refined in bats, the only mammals capable of flapping flight, with the greater spear-nosed bat (*Phyllostomus hastatus)* having the thinnest BGB at 0.1204 *μ*m [23]. Despite the vast range in body mass amongst mammals, the BGB does not appear to be that different, being 0.26 *μ*m in the 2.6 g Etruscan shrew (*Suncus etruscus*) and a close value of 0.35 *μ*m in the bowhead whale (*Balaena mysticetus*), which weighs about 150 tons [24].

The thickest BGB in birds, for which data are available, is found in the flightless species of the ostrich (*Struthio camelus*) leading the pack at 0.56 *μ*m [25], followed by the Humboldt penguin (*Spheniscus humboldti*) at 0.53 *μ*m [26]. In the better studied domestic fowl, the thickness of BGB is intermediate at 0.318 *μ*m. In the emu (*Dromaius novaehollandiae*) [27], a large flightless bird that has evolved in a habitat with few predators, the BGB is much thinner at 0.232 *μ*m.

## 3. Formation of the Mammalian BGB

**Figure 2:**
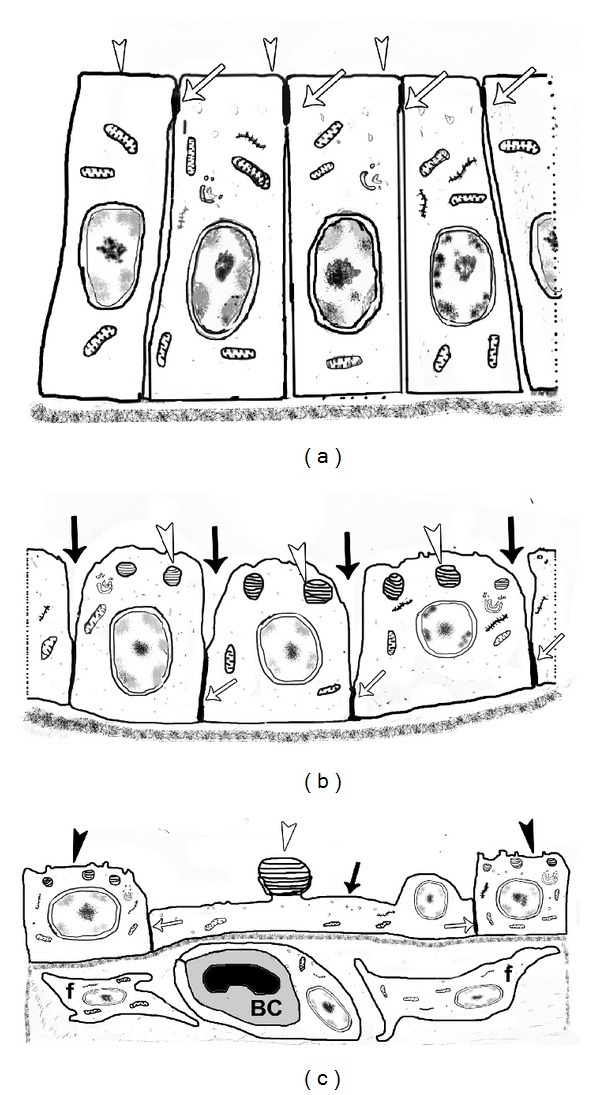
Schematic diagrams showing the steps in attenuation of the epithelium in the mammalian lung. (a) At the pseudoglandular stage, lung tubules are lined with high columnar homogeneous cells with the intercellular tight junctions placed high up towards the tubular lumen (open arrows). Notice also that the cells are devoid of microvilli (open arrowheads). (b) As the epithelium attenuates, cells develop lamellar bodies (open arrowheads) and there is lowering of intercellular tight junctions as the cells become stretched and also the intercellular spaces widen (closed arrows). The epithelial cells at this stage are no longer columnar but cuboidal and the tight junctions have been lowered to the basal part of the epithelium (open arrows). (c) The cells destined to become squamous pneumocytes (AT-I cells) become thinner (closed arrow), extrude their lamellar bodies (open arrowhead) and approximate blood capillaries (BC) so that a thin BGB is formed. Other cells differentiate to ultimate AT-II pneumocytes (closed arrowheads) and have well developed lamellar bodies. Notice also the depressed position of tight junctions (open arrows). Fibroblasts (f) are abundant in the interstitial tissue and are important in laying down collagen.

In mammals, lung development proceeds through well-defined stages chronologically described as embryonic, pseudoglandular, canalicular, saccular, alveolar, and microvascular maturation [6, 12]. The primitive migrating tubes of the pseudoglandular stage are lined by tall columnar cells, which are progressively reduced in height to form the squamous pneumocytes that participate in the formation of the BGB. Initially, the columnar epithelial cells are converted to primitive pneumoblasts containing numerous lamellar bodies [11]. These pneumoblasts later differentiate to definitive AT-I and AT-II cells in the canalicular stage [11, 12, 28]. The majority of these AT-II cells are converted to AT-I cells (Figures 1 and 2), which form the internal (alveolar) layer of the BGB [11, 29]. The conversion of AT-II to AT-I cells entails several events, which include lowering of the intercellular tight junctions between adjacent epithelial cells (Figure 2) such that the apical part of the cells appears to protrude into the lumen [30]. In addition, there is extrusion of lamellar bodies and the cells spread out as the airspaces expand (Figure 1). Subsequent thinning of the cells and ultimate apposition of the blood capillaries [11, 12, 28] accomplish the thin BGB. 

In addition to cell movements, apoptosis of putative superfluous AT-II cells [31] and their subsequent clearance by alveolar macrophages create space for incipient AT-I cells [12]. During the saccular stage, the interalveolar septa have a double capillary system, the epithelium is thick, and the interstitium is abundant, but these are reduced by progressive diminution of the interstitial connective tissue, so that the two capillary layers fuse, resulting in a single capillary of the mature lung [12, 28]. The structure of the BGB in mammals has been described in generous details [19] with the notion that it needs to be extremely thin while maintaining an appreciable strength to withstand stress failure. The basic structure of the BGB has been well conserved through evolution and comprises an epithelium, an interstitium, and an endothelium [32]. 

## 4. Development of the Avian BGB

In birds, the process of BGB formation is totally different from that described in mammals, and lung growth has not been divided into phases. From the laryngotracheal groove formed from the chick primitive pharynx at about 3-4 days of incubation, the primordial lungs arise as paired evaginations. The proximal part of each lung bud forms the extrapulmonary primary bronchus, and the distal one forms the lung. The distal part of the bronchus (mesobronchus) grows into the surrounding mesenchyme and gives rise to the secondary bronchi [8]. The endoderm gives rise to the epithelium of the airway system while the surrounding mesenchymal tissue gives rise to the muscles, connective tissues and lymphatics [8]. Both local vasculogenesis [33] as well as sprouting angiogenesis [34] contributes, to blood vessel formation in the lung. Augmentation, reorganization, and reorientation of the capillaries in forming the thin BGB and the architectural pattern characteristic of the parabronchial unit are by intussusceptive angiogenesis [35].

**Figure 3:**
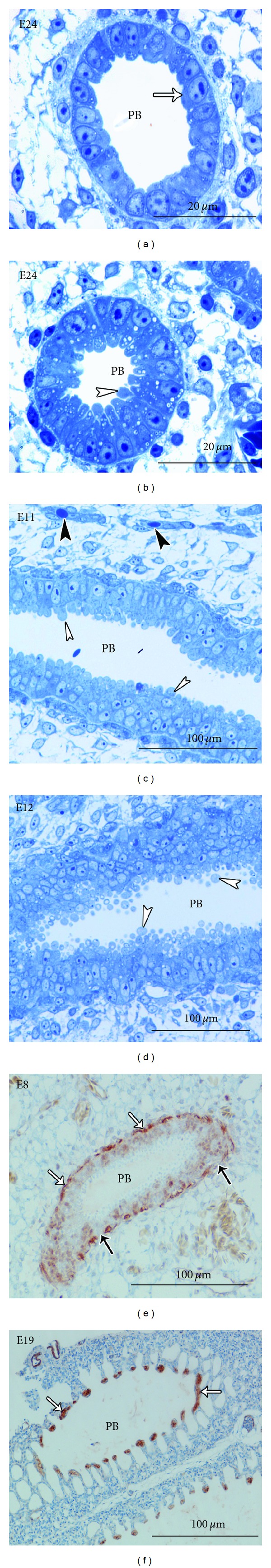
Micrographs from semithin sections ((a)–(d)) showing the coarse changes in the parabronchial epithelium and from paraffin sections showing staining for *α*-smooth muscle actin ((e) and (f)). ((a) and (b)) A close up of individual parabronchial tubes (PB) in the ostrich at E24, showing a cuboidal epithelium (open arrow in (a)) and a thickened columnar epithelium (open arrowhead in (b)). Note that in both cases, the nuclei remain in the basal region; the apical part of the cell becomes elongated thus reducing the parabronchial lumen (PB). ((c) and (d)) By E11 in the chick embryo (c), the parabronchial epithelium is pseudostratified and the apical parts of the cells appear club-like (open arrowheads in (c)). By E12, these apical parts are severed such that they appear to fall off into the parabronchial (PB) lumen (open arrowheads in (d)). Dark arrowheads in (c) show developing capillaries. ((e) and (f)) Chick lung stained for alpha-SMA at E8 and E19 respectively. These alpha-SMA positive cells (open arrows in (e)) surround the developing parabronchus (PB) while leaving some gaps (closed arrowheads in (e)) for future migration of atria. At E19, the atria are well formed and the alpha-SMA positive cells are restricted to the apical parts of the interatrial septa (open arrows in (f)). (a) and (b) are modified from [36] while (c)–(f) are from [15].

**Figure 4:**
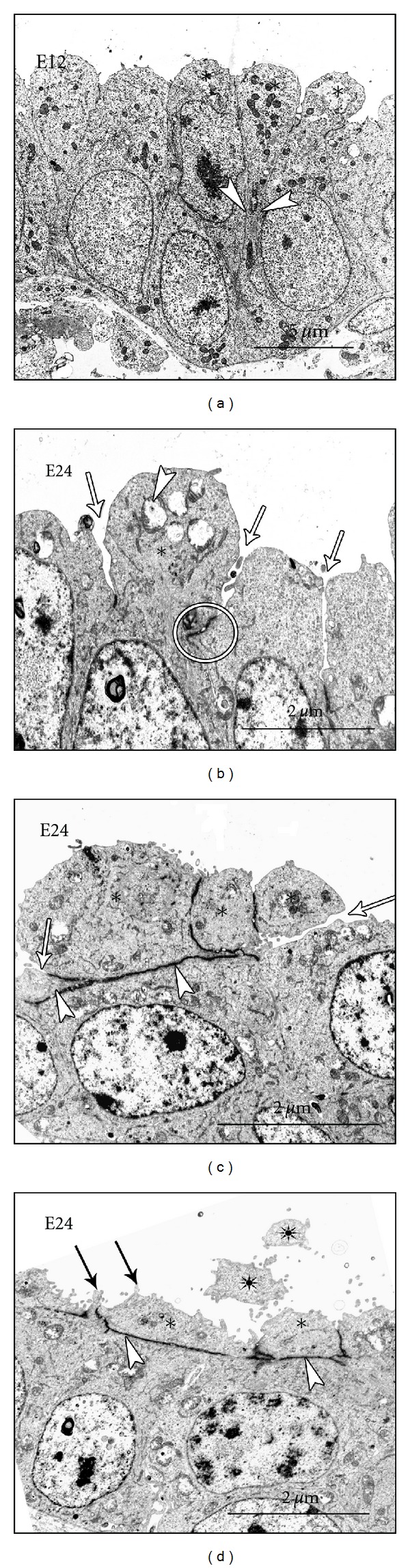
Transmission electron micrographs showing the various stages in attenuation of the avian conduit epithelium. (a) At E12 in the chick embryo, apical elongation of the epithelial cells results in formation of aposomes (stars) and this precedes constriction of the cell at a region below the aposome (arrowheads), due to squeezing by adjacent better endowed cells [37]. (b) In the ostrich embryo at E24 several attenuation processes are evident contemporaneously. In addition to development of lamellar bodies (open arrowhead), there is lowering of tight junctions (open arrows and circle) so that the aposome (star) is clearly delineated. ((c)-(d)) A second method of extruding the aposomes demonstrated in the ostrich involves formation of a double membrane separating the basal part of the cell from the aposome (arrowheads). With subsequent unzipping of the double membrane (open arrows in (c)), the aposome is discharged. Notice the still attached aposomes (stars) and the discharged ones (asterisks in (d)). (b)–(d) are modified from [36]. Closed arrows in (d) indicate microfolds formed after rapture of vesicles.

Formation of the BGB in the chick lung is recognizable at about E8 (E24 in the ostrich) when the cuboidal epithelium is converted to a columnar one, and by E12, it is stratified and shows signs of losing the apical parts (Figure 3). Interestingly, cells positive for *α*-SMA align themselves around the parabronchial tubes leaving gaps for migration of the prospective gas exchanging units. Such cells finally become the smooth muscle cells that support the interatrial septa (Figures 3 and 4). A recent review on the BGB formation in avian embryos [15] has documented what is known, but the information was mainly based on the chicken lung, due to lack of data on other species.

The events in the developing lung of the ostrich closely resemble those of the chick but appear to be delayed by twice the duration (incubation period at 40–42 days is twice that of the chicken). The early events in the ostrich have not been documented, but at E24, the lung resembles that of the chick embryo at embryonic day 8 (E8), with parabronchi lined with a cuboidal to tall columnar epithelium, and some cells are seen to have tapered apical portions and formation of double membranes separating the apical protrusion (aposome) from the basal part of the cell (Figure 3). A detailed description of these cell attenuation processes is only available for the chicken lung [37]. A recent report on the ostrich lung indicates that these events are well conserved in the avian species [36]. For the aforementioned reason, the description herein after is mainly based on the chick lung but is taken to represent the avian species, with specific reference to the ostrich where differences are encountered. 

### 4.1. Peremerecytosis: Cell Decapitation by Constriction or Squeezing

The process of cell attenuation by constriction, strangulation, or even squeezing was dubbed peremerecytosis [37]. Aposome formation by the epithelial cells occurs concomitantly with growth and expansion so that the better endowed cells squeeze out the aposomes of their sandwiched neighbors. Presumably, this results in adherence and subsequent fusion of the lateral membranes of the squeezed cell, and as such the aposome is discharged (Figure 4). Alternatively, aposome formation is followed by the lowering of the tight junctions between adjacent cells then spontaneous constriction of the cell just abovewherethe tight junction occurs. Similar epithelial cell protrusions into the parabronchial lumina were reported in the developing quail lung [38] and in the developing chicken lung [39], but the precise cellular events were not recognized then. The set of diverse morphogenetic events was presented in details on the chicken lung [37], and similar processes have recently been demonstrated in the ostrich [36]. In either case, progressive thinning of the stalk of the protrusion results in severing of the aposome. This process is analogous to aposecretion in exocrine glands [40], the difference being in the contents discharged and the timing of the events. In archetypical aposecretion there is bulging of the apical cytoplasm, absence of subcellular structures, and presence of membrane-bound cell fragments, the so-called aposomes [41].

### 4.2. Secarecytosis: Cell Cutting by Cavitation or Double Membrane Unzipping

The various processes that result in the cutting of the epithelial cells during attenuation have been grouped together under one name, secarecytosis. This terminology describes all the processes that lead to severing of the cell aposome or cell processes such as microfolds without causing constriction. Cutting in this case proceeds through intercellular cavitation or double membrane formation [36, 37]. 

#### 4.2.1. Cell Cutting by Intracellular Space Formation

**Figure 5:**
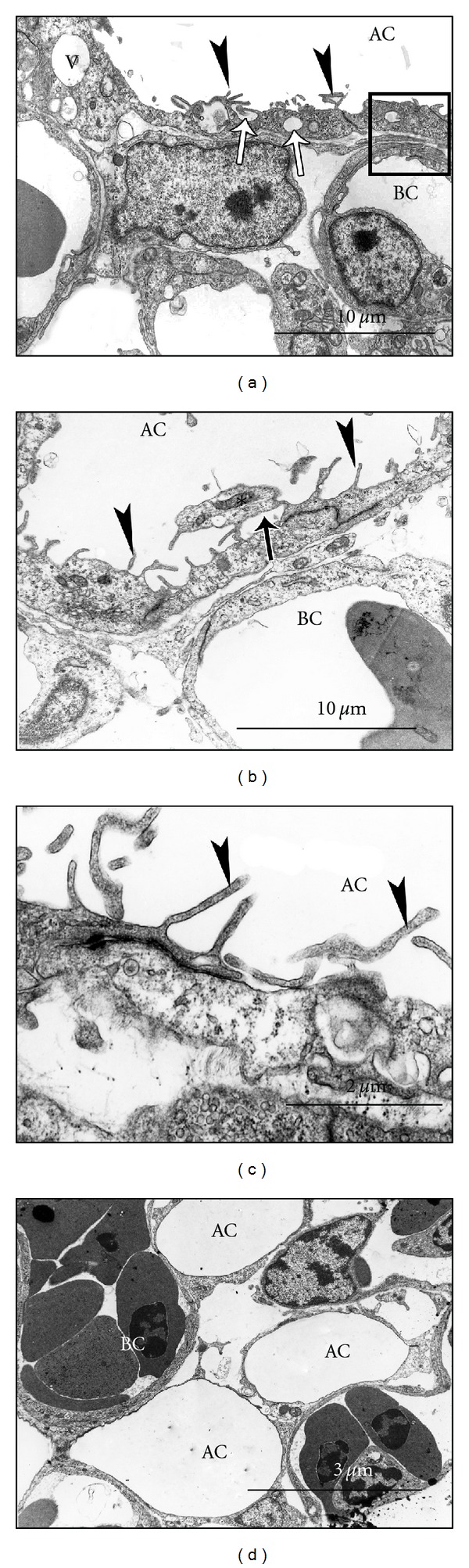
Transmission electron photomicrographs illustrating the additional mechanisms of epithelial attenuation that occur later during the attenuation process in the chiken lung. In all cases the rectangular frame delineates the BGB, BC is blood capillary and AC is air capillary. ((a) and (b)) are modified from [35] while the rest are from [37], with the kind permission of the publisher. ((a) and (b)) Formation of vacuoles (V) and vesicles (open arrows) and their subsequent rapture (fusion with the apical plasmalema) results in formation of numerous microfolds that resemble microvilli (closed arrowheads). Notice an aposome (asterisk in (b)) still attached to the cell apical membrane but still hanging above a vacuole (closed arrow). ((c) and (d)) Microfolds formed as a result of vesicle rapture (closed arrowheads) are severed so that by the time of hatching (d) there were virtually no microfolds. The BGB was similar to that of adults and the air capillaries (AC) were well developed.

The processes and events that preponderate in the later stages of BGB formation in the avian lung have recently been reviewed [15]. Formation of vesicles (endocytic cavities smaller than 50 nm in diameter) or vacuoles (endocytic cavities greater than 50 nm in diameter) in rows below the cell apical portion is seen in later stages of development. Such cavities finally fuse with their neighboring cognates and then with apicolateral plasma membranes and, in doing so, sever the aposomal projection from the rest of the cell in a process referred to as coalescing vesiculation. The latter processes mainly characterize attenuation of the low cuboidal epithelium in the formative atria and infundibulae as well as in the migrating air capillaries. The aposomal bodies released contain abundant organelles and several microfolds. Plausibly, the microfolds result from the fusion of contiguous vesicular/vacuolar membranes at the interphase between the aposome and the basal part of the cell, hence discharging the aposome. The process has been referred to as coalescing vesiculation and is contrasted from rapturing vesiculation where vesicles and vacuoles move towards the apical plasma membrane, fuse with it and discharge their entire contents (Figure 5). The result is that the vacuole remains like a concavity bounded on either side by a microfold that resembles a microvillus on 2D section. If the participating cavities are vacuoles, large folds separating the concavities are formed, while rapture of vesicles leaves tiny microfolds resembling microvilli. Whatever the circumstance, there is concomitant reduction in the cell height. The detailed events were previously reported in the chicken lung [37] and have recently been reported in the ostrich [36].

#### 4.2.2. Cell Cutting by Double Membrane Unzipping

Formation of dark bands across a cell occurs usually between the protruding aposome and the basal part of the cell. The band is believed to be a double plasma membrane, probably associated with cytoskeletal proteins. The double membrane may form the site of separation, whereby the apical part is severed from the basal one (Figure 4). In some cases, the double membrane forms a boundary above which the processes of cell cutting such as rapturing vesiculation take place. These processes have recently been demonstrated in the chicken [37] and the ostrich lungs [36].

## 5. Mechanisms of Epithelial Cell Attenuation

In the mammalian lungs the mechanisms of BGB formation appear rather simple. Lowering of the tight junctions towards the basal part of the cell is followed by stretching of the cell as the airspaces expand. It was, however, noted that in the attenuating cells, there is summary discharge of lamellar bodies (Figures 1 and 2) rather than discharge of the contents [11]. In physiological type II cell secretion, surfactant is discharged through tiny pores averaging 0.2 × 0.4 *μ*m in size on luminal surface of AT-II cells [42]. The details on how exactly the tight junctions are lowered, how the cells become stretched, or even how the entire lamellar bodies are squeezed out are lacking.

The processes and mechanisms involved in attenuation of the epithelium of the chicken lung are much more complicated but to a large extent resemble physiological secretory processes. In general, they lead to progressive reduction in the cell height until the required thickness is attained. As observed in the developing chicken lung, the primitive tubes at E8 are mainly lined by cuboidal epithelium, which converts to high columnar, then becomes stratified columnar with the onset of the first signs of attenuation (Figure 3). Subsequently, the epithelium undergoes dramatic size reduction and loses morphological polarization by the processes described above. These processes closely resemble aposecretion, where a portion of a cell is discharged with its contents, minus the organelles. 

During aposecretion, proteins such as myosin and gelsolin [43] or even actin [44, 45] have been implicated in extrusion of the apical protrusions. Presence of actin filaments in the constricting aposome has been demonstrated in the attenuating epithelium of the chick embryo lung, plausibly implicating it in the cell cutting process [37]. The actin filaments were localized at the level of the aposomal constriction since they are associated with the cell adhesion belt [46]) and are also indicators for distal relocation of cell junctions [37]. Change of shape in ingressing embryonic cells has been reported. The apices of such cells are constricted, plausibly through actinomyosin contraction [47] with the result that organelles are displaced basally in readiness for migration. Over and above the actinomyosin activity, physiological aposecretion as occurs in the reproductive system, is also driven by hormones and muscarinic receptors [43].

Smooth muscle cells staining positively for alpha actin have been shown to be associated with the developing parabronchi in the chicken lung. Notably, such cells become aligned at the basal aspects of parabronchial epithelial cells delineating gaps through which incipient atria sprout (Figure 3). The *α*-SMA-positive cells, while playing a role in tubular patterning, may be important in epithelial attenuation. During milk secretion, for example, myoepithelial cells below the secretory epithelium squeeze the epithelial cells above and, in so doing, facilitate the release of milk into the secretory acinus [48]. Plausibly, association of *α*-SMA-positive cells with the attenuating air conduit epithelium during epithelial attenuation is important in facilitating such aposecretion-like cell processes. 

## 6. Physiological Adaptation and Remodeling of the BGB

The pulmonary BGB undergoes certain changes that include increase in the thickness of the basement membranes and breaks in the endothelium as a result of stress failure [1, 2]. Continual regulation of the wall structure of the BGB occurs through rapid changes in gene expression for extracellular matrix proteins and growth factors in response to increases in capillary wall stress. This helps to maintain the extreme thinness with sufficient strength [49].

Structural alterations in the BGB in response to physiological changes have been demonstrated. Berg and co-workers [50] subjected lungs to high states of inflation over 4 hours with the result that gene expression for *α*1(III) and *α*2(IV) procollagens, fibronectin, basic fibroblast growth factor (bFGF), and transforming growth factor *β*1 (TGF-*β*1) were increased. Similarly, Parker and colleagues increased venous pressure in perfused isolated rabbit lungs with the finding that there was significant increase in mRNA for *α*1(I) but not *α*2(IV) procollagen [51]. The difference was thought to be because both experimental techniques increase stress in structures other than capillaries. In young dogs subjected to prolonged low oxygen tensions (high altitude), there was notable reduction in harmonic mean thickness of the BGB and a shift in its frequency distribution such that thinner segments were more preponderant [52]. This indicates redistribution of tissue components within the alveolar septa in such a way that there is minimized diffusive resistance.

Breaks in the BGB in cases of extreme stress have been reported. In thoroughbred racehorses after galloping, excessive pressures can lead to pulmonary capillary failure with the resultant pulmonary hemorrhage [53]. In related studies, increase in red blood cells and protein in the broncho-alveolar lavage fluid of exercising elite athletes indicated that the integrity of the blood-gas barrier is impaired by short-term exercise [54]. Similar findings were documented from a rabbit model of increased capillary pressure with subsequent damage to all or parts of the blood-gas barrier [55]. The lack of significant elevations in the cytokines known to increase the permeability of the capillary endothelium mitigates against an inflammatory mechanism and supports the hypothesis that mechanical stress may impair the function of the human blood-gas barrier during exercise [54]. Extremely high stress in the walls of the pulmonary capillaries, as may occur in mechanical ventilation, results in ultrastructural changes including disruptions of both the alveolar epithelial and capillary endothelial layers [56]. Stress failure can result from pathological conditions that interfere with its structural and/or physiological integrity. Such conditions include high-altitude pulmonary edema, neurogenic pulmonary edema, severe left ventricular failure, mitral valve stenosis, and overinflation of the lung [56]. There is a spectrum of low permeability to high permeability edema as the capillary pressure is raised. Remodeling of pulmonary capillaries apparently occurs at high capillary pressures. It is likely that the extracellular matrix of the capillaries is continuously regulated in response to capillary wall stress.

## 7. Molecular Regulation of BGB Development 

A detailed discussion of molecular control of BGB formation needs to consider the various coarse components that come into play during its establishment. On the vascular side is the capillary endothelium, the middle layer is the extracellular matrix (ECM), while the epithelium lines the airspaces. Recently, Herbert and Stainier [57] have provided an updated review of the molecular control of the endothelial cell differentiation, with the notion that VEGF and Notch signaling are important pathways. Angiogenesis itself is a complex process which is currently under intensive investigation and whose molecular control is slowly falling into shape [58]. The intermediate layer of the BGB starts by being excessively abundant but is successfully diminished and, in doing so, the capillary endothelium approximates the attenuating gas exchange epithelium. Therefore, the genes that come into play in production and regulation of the matrix metalloproteinases the enzymes that lead to reduction in ECM are important in lung development [59] and BGB formation. Detailed reports of the molecular control of angiogenesis and ECM biosynthesis are, however, not within the scope of the current discussion, and we will concentrate on differentiation of the alveolar/air capillary epithelium and its subsequent approximation to the endothelium.

The lung in vertebrates is known to be compliant except in avian species. Therefore, some commonalities would be expected in the inauguration and early stages of lung development. Lung development has been well studied in mammals and to some reasonable extent in birds, but not much has been done in the ectotherms. Reports on the reptilian lung structure [22, 60, 61] and in the frog [62] and fish [5] indicate that the parenchymal interairspace septa do not mature, and a double capillary system is retained in these ectotherms. While controlling molecules may be similar to those in mammals and birds at the inaugural stages of lung development, subtle differences would be expected when it comes to later stages of lung maturation. Indeed, many of the controlling factors have been highly conserved through evolution [7, 32].

 Lung development is driven by two forces: intrinsic factors that include a host of regulatory molecules and extrinsic forces, the main one being extracellular lung fluid [63]. A complex set of morphoregulatory molecules constitutes the intrinsic factors, which can be grouped into three classes: transcription factors (e.g., Nkx2.1 also known as thyroid transcription factor-1 (TTF-1), GATA, and HNF-3); signaling molecules such as FGF, BMP-4, PDGF, Shh, and TGF*-*β**; extracellular matrix proteins and their receptors [7, 63, 64]. In mammals, extrinsic/mechanical forces have been shown to be important for fetal alveolar epithelial cell differentiation. Such forces emanate from fetal lung movements that propel fluid through incipient air conduits [65]. 

Formation of BGB in mammals involves the attenuation of the developing lung epithelium, which includes conversion of the columnar epithelium of the pseudoglandular stage to a mainly cuboidal one with lamellar bodies (Figure 1). Subsequently, there is a lowering of the intercellular tight junctions, spreading or stretching of the cell, and total extrusion of lamellar bodies (Figure 2) leading to differentiated AT-I and AT-II epithelial cells. The AT-I cells constitute a thin squamous epithelium that covers over 90% of the alveolar surface area, which provides gas exchange between the airspaces and pulmonary capillary vasculature. AT-II are interspersed throughout the alveoli and are responsible for the production and secretion of pulmonary surfactant, regulation of alveolar fluid homeostasis, and differentiation into AT-I cells during lung development and injury. Genetic control of the specific aforementioned steps has not been investigated, but there exists reports on the differentiation of AT-II and AT-I cells and conversion of AT-II to AT-I cells in mammals [66]. Some of the molecular signals that have been proposed to be involved in the differentiation of AT-II and AT-I cells are (i) transcription factors such as thyroid transcription factor-1 (TTF-1), forkhead orthologs (FOXs), GATA6, HIF2*α*, Notch, glucocorticoid receptor, retinoic acid, and ETS family members; (ii) growth factors such as epithelial growth factor (EGF) and bone morphogenetic protein 4 (BMP4); (iii) other signaling molecules including connexin 43, T1 alpha, and semaphorin 3A. Herein after, the role of these molecules in epithelial cell differentiation in the distal lung is briefly described. 

### 7.1. Molecular Regulation of BGB in Mammals

#### 7.1.1. Growth Factors

*(1) ErbB Growth Factor Receptors.* Growth factors regulate the growth and development of the lung. Growth factors signal their mitogenic activities through tyrosine kinase receptors. Epithelial growth factor receptor (EGFR), a member of the ErbB transmembrane tyrosine kinases, and its ligand (epithelial growth factor, EGF) have been shown to be involved in alveolar maturation. EGF deficiency in rats during perinatal development using EGF autoantibodies results in mild respiratory distress syndrome and delayed alveolar maturation [67]. Inactivation of EGFR/ErbB1 by gene targeting in mice resulted in respiratory failure as a result of impaired alveolarization including presence of collapsed [68] or thick-walled alveoli [68]. EGFR is also important for the AT-II differentiation as lungs from EGFR^−/−^ mice have decreased expression of AT-II specification markers, surfactant proteins (SP)–B, C, and D [69]. ErbB4, another member of the ErbB receptors family, has been also shown to be involved in alveolar maturation. Deletion of ErbB4 in mice results in alveolar hypoplasia during development and hyperreactive airways in adults. Moreover, developing lungs from ErbB4^−/−^ mice exhibited impaired differentiation of AT-II cells with decreased expression of SP-B and decreased surfactant phospholipid synthesis, indicating that ErbB4 plays a role in the differentiation of AT-II cells [70]. Recently, it has been demonstrated that EGFR and ErbB4 regulate stretch-induced differentiation of fetal type II epithelial cells via the ERK pathway [69]. 

*(2) Bone Morphogenetic Protein 4 (BMP4).* Bone morphogenetic protein 4 (BMP4), a transforming growth factor-*β* (TGF*β*), is highly expressed in the distal tips of the branching lung epithelium, with lower levels in the adjacent mesenchyme. The role of BMP4 in alveolar differentiation has been examined by using transgenic mice that overexpress BMP4 throughout the distal epithelium of the lung using the SP-C promoter/enhancer. The BMP4 transgenic lungs are significantly smaller than normal, with greatly distended terminal buds at E16.5 and E18.5 and at birth contain large air-filled sacs which do not support normal lung function [71]. Furthermore, whole-mount *in situ* hybridization analysis of BMP4 transgenic lungs using probes for the proximal airway marker, CC10, and the distal airway marker, SP-C, showed normal AT-II differentiation of bronchiolar Clara cells but a reduction in differentiated cells, indicating that BMP4 plays an essential role in the alveolar epithelial differentiation [71].

#### 7.1.2. Transcription Factors and Nuclear Receptors

*(1) GATA-6 Transcription Factor.* Expression of GATA-6, a member of the GATA family of zinc finger transcription factors, occurs in respiratory epithelial cells throughout lung morphogenesis. Dominant negative GATA-6 expression in respiratory epithelial cells inhibits lung differentiation in late gestation and decreases expression of aquaporin-5, the specific marker for AT-I, and surfactant proteins [70] often acting synergistically with TTF-1 [72]. Overexpression of GATA-6 in the epithelium was shown to inhibit alveolarization, and there was lack of differentiation of AT-II and AT-I epithelial cells as well as failure of surfactant lipid synthesis [70]. In mice expressing increased levels of GATA-6 in respiratory epithelial cells, postnatal alveolarization was disrupted, resulting in airspace enlargement.

*(2) Forkhead Orthologs (FOXs) Transcription Factors.* Foxa1 and Foxa2, members of the winged-helix/forkhead transcription family, are expressed in the epithelium of the developing mouse lung and are important for epithelial branching and cell differentiation. Mice null for Foxa1 do not develop squamous pneumocytes, and although the pulmonary capillaries are well developed, no thin BGB is formed [73]. Previously, it was demonstrated that Foxa2 controls pulmonary maturation at birth. Neonatal mice lacking Foxa2 expression develop archetypical respiratory distress syndrome with all of the morphological, molecular, and biochemical features found in preterm infants, including atelectasis, hyaline membranes, and the lack of pulmonary surfactant lipids and proteins, and they die at birth [74].

*(3) Thyroid Transcription Factor (TTF-1).* The transcription factor TTF-1, a member of the Nkx homeodomain gene family, is expressed in the forebrain, thyroid gland, and lung. In the lung, TTF-1 plays an essential role in the regulation of lung morphogenesis and epithelial cell differentiation via transactivating several lung-specific genes including the surfactant proteins A, B, C, D, and CC10 [75]. Mice harboring a null mutation in the TTF-1 gene exhibit severely attenuated lung epithelial development with a dramatic decrease in airway branching. Moreover, lung epithelial cells in these mice lack expression of SP-C suggesting that TTF-1 is the major transcription factor for lung epithelial gene expression [76]. Mutations in the human TTF-1 gene have been associated with hypothyroidism and respiratory failure in human infants [77]. 

*(4) Hypoxia-Inducible Factor 2*α* (HIF2*α*).* Hypoxia-inducible factor 2*α* (HIF2*α*), an oxygen-regulated transcription factor, in the lung is primarily expressed in endothelial, bronchial, and AT-II cells. The role of HIF2*α* in AT-II cells was examined by using transgenic mice that conditionally expressed an oxygen-insensitive mutant of HIF2*α* (mutHIF2*α*) in airway epithelial cells during development [69]. These mice were shown to have dilated alveolar structures during development, and the newborn mice died shortly after birth due to respiratory distress. Moreover, the distal airspaces of mutHIF2*α* lungs contained abnormal morphology of AT-II cells including an enlarged cytoplasmic appearance, a decreased formation of lamellar bodies, and a significantly reduced number of AT-I cells with decreased expression of aquaporin-5. Therefore, it was indicated that HIF2*α* negatively regulates the differentiation of AT-II to AT-I cells. Inactivation of HIF2*α* in transgenic mice resulted in fatal respiratory distress in neonatal mice due to insufficient surfactant production by AT-II cells. Furthermore, lungs of HIF2*α* ^−/−^mice exhibited disruption of the thinning of the alveolar septa and decreased numbers of AT-II cells, indicating that HIF2*α* regulates the differentiation of AT-II cells [78].

*(5) Notch.* Notch signaling is also involved in the differentiation of AT-II cells to AT-I cells in mammals. Overexpression of Notch1 in the lung epithelium of transgenic mice constitutively expressing the activation domain (NICD) of Notch 1 in the distal lung epithelium using a SP-C promoter/enhancer prevented the differentiation of the alveolar epithelium [79]. In these mice, lungs at E18.5 had dilated cysts in place of alveolar saccules. The cysts composed of cells that were devoid of alveolar markers including SP-C, keratin 5, and p63, but expressed some markers of proximal airway epithelium including E-cadherin and Foxa2. Thus, Notch1 arrests differentiation of alveolar epithelial cells. Notch3, another member of the Notch signaling pathway, has also been demonstrated to play a role in alveolar epithelial differentiation. Transgenic mice that constitutively express the activated domain of Notch3 (NICD) in the distal lung epithelium using a SP-C promoter/enhancer were shown to be embryonic lethal at E18.5 and harbored altered lung morphology in which epithelial differentiation into AT-I and AT-II cells was impaired. Metaplasia of undifferentiated cuboidal cells in the terminal airways was also evident [80]. Therefore, constitutive activation of Notch3 arrests differentiation of distal lung alveolar epithelial cells. Recent complementary evidence showed that pharmacological approaches to disrupt global Notch signaling in mice lung organ cultures during early lung development resulted in the expanding of the population of the distal lung progenitors, altering morphogenetic boundaries and proximal-distal lung patterning [81]. 

*(6) Glucocorticoid Receptor and Retinoic Acid.* Glucocorticoids are important for the maturation of the fetal lung, and glucocorticoid actions are mediated via the intracellular glucocorticoid receptor (GR), a ligand-activated transcriptional regulator. The role of glucocorticoid action via GR signaling in fetal lung maturation has been demonstrated by using GR-null mice [82]. The lungs of fetal GR-null mice were found to be hypercellular with blunted septal thinning leading to a 6-fold increase in the airway to capillary diffusion distance and hence failure to develop a functionally viable BGB [82]. The phenotype of these mice was accompanied with increased number of AT-II cells and decreased number of AT-I cells with decreased mRNA expression of AT-I specific markers T1 alpha and aquaporin-5. The conclusion in these studies was that receptor-mediated glucocorticoid signaling facilitates the differentiation of epithelial cells into AT-I cells but has no effect on AT-II cell differentiation.

Retinoic acid receptor (RAR) signaling is important early during development but its role has a temporal disposition. RAR signaling establishes an initial program that assigns distal cell fate to the prospective lung epithelium. Downregulation of RA signaling in late prenatal period is requisite for eventual formation of mature AT-I and AT-II cells [82, 83]. Furthermore, RAR activation interferes with the proper temporal expression of GATA6, a gene that is critical in regulation of surfactant protein expression in branching epithelial tubules and establishment of the mature AT-II and AT-I cell phenotypes [84]. Later during lung development, RAR signaling is essential for alveolar formation [85].

*(7) E74-Like Factor 5 (ELF5).* E74-like factor 5 (ELF5), an Ets family transcription factor, is expressed in the distal lung epithelium during early lung development and then becomes restricted to proximal airways at the end of gestation. Overexpression of ELF5, specifically in the lung epithelium during early lung development by using a doxycycline inducible HA-tagged ELF5 transgene under the SP-C promoter/enhancer, resulted in disrupted branching morphogenesis and delayed epithelial cell differentiation [86]. Lungs overexpressing ELF5 exhibited reduced expression of the distal lung epithelial differentiation marker SP-C [86], indicating that ELF5 negatively regulates AT-II differentiation.

*(8) Wnt/*β*-catenin.* The Wnt/*β*-catenin pathway regulates intracellular signaling, gene transcription and cell proliferation and/or differentiation. The essential role of the Wnt/*β*-catenin pathway in the differentiation of alveolar epithelium has been demonstrated by using transgenic mice in which *β*-catenin was deleted in the developing respiratory epithelium, using a doxycycline-inducible conditional system to express Cre recombinase-mediated, homologous recombination strategy [87]. Deficiency of *β*-catenin in the respiratory epithelium resulted in pulmonary malformations consisting of multiple, enlarged, and elongated bronchiolar tubules and disruption of the formation and differentiation of distal terminal alveolar saccules, including the specification of AT-I and AT-II epithelial cells in the alveolus [87].

#### 7.1.3. Other Molecular Signals

*(1) Semaphorin 3A.* Semaphorin 3A (Sema3A), a neural guidance cue, mediates cell migration, proliferation, and apoptosis and inhibits branching morphogenesis. The role of Sema3A in maturation and/or differentiation of the distal lung epithelium during development was deduced from studies on Sema3A-null mice. Lungs from Sema3A^−/−^ embryos had reduced airspace size and thickened alveolar septae with impaired epithelial cell maturation of AT-I and AT-II cells [88].

*(2) Connexin 43.* Connexin 43, one of the connexins family members that form gap junctions, is one of the most studied proteins in organogenesis. During early lung branching morphogenesis in mice, connexin 43 is highly expressed in the distal tip endoderm of the embryonic lung at E11.5, and after birth, connexin-43 is expressed between adjacent AT-I cells in rats and mice. Connexin 43 knockout mice die shortly after birth due to hypoplastic lungs [89]. Lungs from connexin 43^−/−^ mice exhibit delayed formation of alveoli, narrow airspaces, and thicker interalveolar septae. Additionally, such lungs have decreased mRNA expressions of AT-II specific marker SP-C gene, AT-I specific marker aquaporin-5, and *α*-SMA actin and have reduced numbers of AT-I cells [89].

*(3) T1 Alpha.* T1 alpha, a differentiation gene of AT-I cells, is highly expressed in the lung at the end of gestation. T1 alpha is only expressed in AT-I cells but not AT-II cells. Evidence for participation of T1 alpha in differentiation of AT-I cells but not AT-II cells was adduced from studies on knockout mice. Homozygous T1 alpha null mice die at birth due to respiratory failure, and lungs exhibit abnormal high expression of proliferation markers in the distal lung [81]. There is normal differentiation of AT-II cells with normal expression of surfactant proteins, lack of differentiation of AT-I cells with decreased expression of aquaporin-5, narrower and irregular airspaces, and defective formation of alveolar saccules. Comparison of microarray analyses of T1 alpha^−/−^ and wild-type lungs showed that there was an altered expression of genes including upregulation of the cell-cell interaction gene ephrinA3 and downregulation of negative regulators of the cellcycle such as FosB, EGR1, MPK-1 and Nur11 [90]. 

### 7.2. Molecular Regulation of BGB Formation in Birds

The avian lung differs fundamentally from that of other vertebrates in having noncompliant terminal gas exchange units. While the upstream control of lung development may be close or similar to that of the other vertebrates, later events indicate that a totally different process occurs. Formation of the BGB requires that the blood capillaries (BCs) and the attenuating air capillaries (ACs) migrate through progressively attenuating interstitium to approximate each other [35, 37]. Elevation of levels of basic FGF (bFGF), VEGF-A, and PDGF-B during the later phase of avian lung microvascular development [34] indicated that they may be important during interaction of the BCs and the ACs. In the chicken lung, pulmonary noncanonical Wnt5a uses Ror2 to control patterning of both distal airway and vascular tubulogenesis and perhaps guides the interfacing of the air capillaries with the blood capillaries [91]. The latter authors showed that lungs with mis-/overexpressed Wnt5a were hypoplastic with erratic expression patterns of Shh, L-CAM, fibronectin, VEGF, and Flk1. Coordinated development of pulmonary air conduits and vasculature is achieved through Wnt5a, which plausibly works through fibronectin-mediated VEGF signaling through its regulation of Shh [91]. Fibroblast growth factors (FGFs) and their cognate receptors (FGFRs) are expressed in the developing chick lung and are essential for the epithelial-mesenchymal interactions. Such interactions determine epithelial branching [92] and may be essential for ultimate BGB establishment. 

## 8. Conclusion

In the current paper, we have presented an overview of the events that take place during inauguration, development, and remodeling of the vertebrate BG. We have highlighted the fact that the events differ fundamentally between the compliant mammalian lung and the rigid avian lung. The paper is skewed towards the formation of the internal (alveolar/air capillary) layer of the BGB. Specific studies on molecular control of BGB formation are lacking, but investigations on the AT-II and AT-I cell differentiation in mammals exist. While there is a rapidly increasing wealth of studies on molecular control of the mammalian lung development, very little has been done on the avian species. Studies on the factors guiding and controlling the newly described cell processes of secarecytosis and peremerecytosis in the avian lung are strongly recommended. Furthermore, investigations focused on epithelial attenuation and epithelial-endothelial interactions would illuminate the mechanisms preponderant during BGB formation.
